# Writer’s cramp as a presentation of L-2-hydroxyglutaric aciduria

**DOI:** 10.1186/s40734-014-0009-9

**Published:** 2014-12-11

**Authors:** Pichet Termsarasab, Steven J Frucht

**Affiliations:** Movement Disorder Division, Department of Neurology, Icahn School of Medicine at Mount Sinai, 5 East 98th St, New York, 10029 NY USA

**Keywords:** L-2-hydroxyglutaric aciduria, Dystonia, Writer’s cramp, Movement disorders, Metabolic disease

## Abstract

L-2-hydroxyglutaric aciduria (L2HGA) is a neurometabolic disorder characterized by macrocephaly, seizures, progressive mental retardation, pyramidal signs, ataxia and tremor. Dystonia is an under-recognized feature of this entity in the literature. We report two siblings with L2HGA, one of whom presented with writer’s cramp followed by dystonia of the other hand. An elevated plasma lysine, highly elevated urine 2-hydroxyglutaric acid, and MRI with characteristic findings (leukoencephalopathy of bilateral subcortical white matter sparing central white matter) suggested the diagnosis, which was confirmed by genetic testing.

**Electronic supplementary material:**

The online version of this article (doi:10.1186/s40734-014-0009-9) contains supplementary material, which is available to authorized users.

## Background

L-2-hydroxyglutatric aciduria (L2HGA) is a neurometabolic disorder in the family of cerebral organic acid disorders, examples of which include glutaric aciduria type I, saccharopinuria, 2-amino-/2-oxo-adipic aciduria, D-2-hydroxyglutaric aciduria and Canavan’s disease (N-acetylaspartic aciduria) [1]. L2HGA is an autosomal recessive disorder with the defective gene encoding a Flavin Adenine Dinucleotide (FAD)-dependent L-2-hydroxyglutarate dehydrogenase. The typical clinical presentation includes macrocephaly and progressive neurologic signs such as febrile and afebrile seizures, cerebellar deficits (gait and limb ataxia), speech problems, learning disabilities and developmental delay, and pyramidal signs [2]. Movement disorders described in the literature in these patients include tremor, dystonia and ataxia [2]-[4]. We present two siblings with L2HGA, one of whom presented with focal hand dystonia, to call attention to this unusual condition.

## Case presentation

### Case 1

The proband is a 15-year-old Egyptian girl. She developed difficulty writing with her right hand at age 8. The problem started selectively with writing but soon spread to involve other tasks. She switched to writing with her left hand, and developed writing problem two years later. Subsequently she developed problems with other tasks including holding utensils and using a computer for school. She had slow developmental milestones and some cognitive impairment but no history of seizures. Her ethnic background is Egyptian/Turkish and Egyptian/Syrian on maternal and paternal sides respectively, with no known consanguinity.

**Figure 1:**
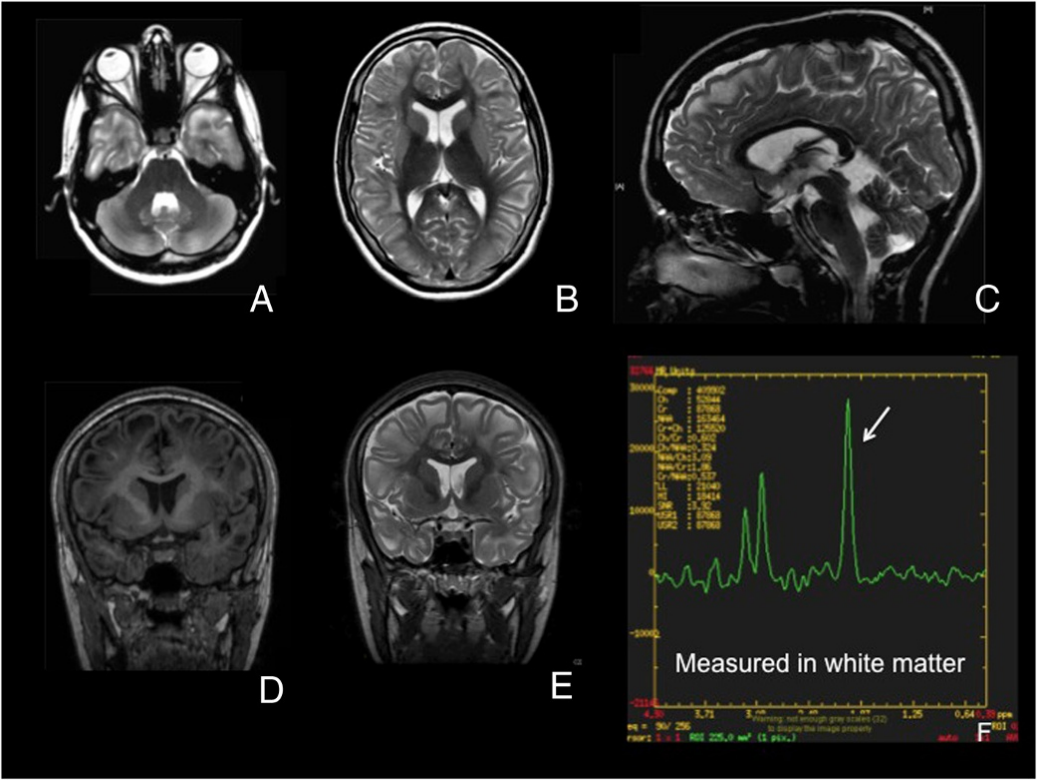
**Panels A to E reveal the MRI of patient 1. A** and **B**, axial sections of T2-weighted images (T2WI); **C**, mid-sagittal plane of T2WI; **D** and **E**, coronal sections of T1WI and T2WI, respectively; **F**, MR spectroscopy of voxel at subcortical white matter. MRI shows white matter changes in subcortical region diffusely, seen as hypointense and hyperintense signal on T1WI and T2WI, respectively. There is sparing of central white matter. There is also T2-hyperintense signal at bilateral dentate nuclei (panel **A**).

Physical examination revealed no macrocephaly. Eye movements were normal, and her speech was slightly scanning but not dysarthric. As soon as she picked up a pen to write, there was moderate to severe action dystonia in both arms, more prominent on the left. There was no null point or geste antagoniste (Figure 1 and Additional file 1). There was no rest or action tremor. There was mild dysmetria on finger-to-nose testing. Gait was normal.

**Figure 2:**
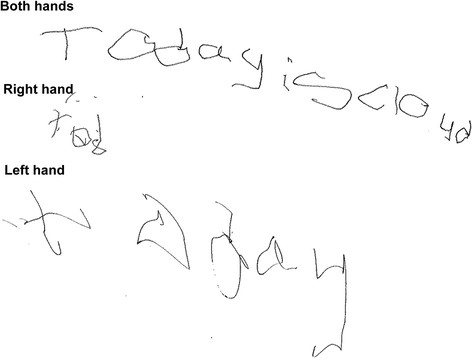
**Handwriting of patient 1, when on trihexyphenidyl 2 mg/day.** The upper, middle and lower rows demonstrate handwriting when she was allowed to write with only the right or the left hand, respectively. Her handwriting was worse with the right hand, correlated with the degree of dystonia in each hand.

Biochemical testing revealed elevated plasma lysine and highly elevated urine 2-hydroxyglutaric acid. Molecular genetic testing showed a heterozygous mutation in the *L2HGDH* gene – c.584A>G and c.1115delT. MRI of the brain revealed marked diffuse subcortical white matter abnormalities with hypointense and hyperintense signals on T1 and T2 images, and relative sparing of central white matter. There were also hyperintense signals in both dentate nuclei on T2 images (Figure 1A-E). MR spectroscopy revealed a high lactate peak and reduced N-acetylaspartate to creatine ratio (Figure 1F). She was treated with trihexyphenidyl up to 6 mg/day, but could not tolerate it due to the side effect of worsening cognition. The dose was reduced to 2 mg/day (Figure 2). She also takes riboflavin.

### Case 2

The sister of patient one is sixteen. She had delayed developmental milestones starting in early childhood, but was not tested until her younger sister manifested the symptoms. On examination, she had mild kinetic tremor of both hands, and finger-to-nose test showed mild past-pointing. Her speech was mildly scanning with no dysarthria. She could not perform tandem walking. There was no evidence of dystonia.

MRI of the brain showed findings similar to the proband, and genetic testing also revealed the same mutation as the proband. She is also on riboflavin but on no medication for tremor.

## Discussion

The diagnosis of L2HGA is usually considered when plasma amino acid and urine organic screening tests show elevated lysine and 2-hydroxyglutaric acid, respectively. We suspect that few movement disorder neurologists would consider L2HGA in a young patient with writer’s cramp. Indeed, lists of causes of secondary dystonia in major movement disorder textbooks do not even include this condition.

The phenomenology of movement disorders in L2HGA reported in the literature includes dystonia, tremor, ataxia and rarely myoclonus. Dystonia has been described affecting the hands and upper limbs [4]. Tremor described in the literature is mostly intention tremor, similar to the intention tremor of case 2. Ataxia can affect either the trunk or limbs [2], and mild myoclonus in the upper extremity has been reported in one patient [2].

Although the most common initial presentation reported by Barth and colleagues were walking delay and gait abnormality (6 out of 8 patients), movement disorders may be under-recognized [2]. Balaji et al. recently described 2 siblings with L2HGA, one of which with dystonia of the head, trunk and upper extremities, the other with dystonia of distal upper and lower extremities [5]. In our patient (proband), the initial presentation that prompted her parents to bring her to medical attention was writer’s cramp. Focal task-specific dystonia of the arm is unusual in childhood outside of the setting of DYT-1 dystonia. It has also been reported in myoclonus-dystonia syndrome (DYT-11) [6],[7] and X-linked dystonia-parkinsonism (DYT-3). Writer’s cramp has been reported as a presentation in a limited number of secondary dystonias in children such as basal ganglia arteriovenous malformations [8], basal ganglia infarction [9], Wilson’s disease, Huntington’s disease, pantothenate kinase-associated neurodegenration (PKAN), glutaric aciduria, GM1 gangliosidosis, and Leigh’s disease. L2HGA should be added to the list of secondary dystonias of childhood.

In the Krebs cycle, 2-ketoglutaric acid is usually catalyzed by the enzyme L-malate dehydrogenase to L-2-hydroxyglutaric acid. It has been proposed that L-2-hydroxyglutaric acid is an unwanted substrate which then requires a “repair” enzyme, L-2-hydroxyglutaric acid dehydrogenase, to shift it back to 2-ketoglutaric acid in the Krebs cycle [10]. This “repair” enzyme is deficient in L2HGA. MRI findings in this disorder are unique, and once seen, are pathognomonic. The neuroradiologic differential diagnoses of leukoencephalopathy with grey matter signal abnormalities (with neuroanatomical structures involved in parenthesis) include Canavan’s disease (globus pallidi, dentate nuclei), L2HGA (basal ganglia, dentate nuclei), Alexander’s disease (deep grey matter, dentate hilum), GM1/GM2 gangliosidoses (basal ganglia), Krabbe’s disease (thalami, dentate nuclei), and maple syrup urine disease (thalami, globus pallidi) [11].

Although treatment options are limited in L2HGA, it is important to recognize this disorder as it is associated with malignant brain tumors such as glioblastoma and primitive neuroectodermal tumor [12], requiring close surveillance. Riboflavin treatment [13] and protein restriction may have a role, but data is limited. FAD and levocarnitine chloride were also reported as treatment options [14].

An animal model of L2HGA has been described in Staffordshire Bull Terriers, with seizures, ataxia, tremor, and dementia [15],[16].

## Conclusion

It is important to have a high index of suspicion of secondary dystonia when evaluating pediatric patients who initially present with upper extremity dystonia or writer’s cramp. We illustrate the example of this phenotype as an initial presentation of L2HGA.

## Consent

Written informed consent was obtained from the patient for publication of this case report and any accompanying images. A copy of the written consent is available for review by the Editor-in-Chief of this journal.

## Additional file

## Electronic supplementary material

Additional file 1: **Video 1.** The speech of patient 1 was normal. There was dystonia of bilateral hands, left greater than right, with spooning of the fingers. She did not have tremor or ataxia. During writing, there was moderate extension of the right wrist and abduction of the right elbow. There was no mirror dystonia when she wrote with the left hand. There was excessive bilateral arm swing during walking. The latter segment of the video shows examination 3 months after she was treated with trihexyphenidyl 6 mg/day. She preferred to write with both hands. Writer’s cramp was slightly improved from the previous examination when she was allowed to write with only the right hand. Right focal hand dystonia was also mildly better when she held the pen between her right index and middle fingers. There was mild flexion of the left wrist when she wrote with her left hand. The dose of trihexyphenidyl was subsequently reduced due to cognitive side effects. (ZIP 11 MB)

Below are the links to the authors’ original submitted files for images.

Authors’ original file for figure 1

Authors’ original file for figure 2
