# Supplementary figures and images for: Writer’s cramp as a presentation of L-2-hydroxyglutaric aciduria
Source: J Clin Mov Disord. 2014 Dec 11;1:9. doi: 10.1186/s40734-014-0009-9 (PMC4711040; doi:10.1186/s40734-014-0009-9)

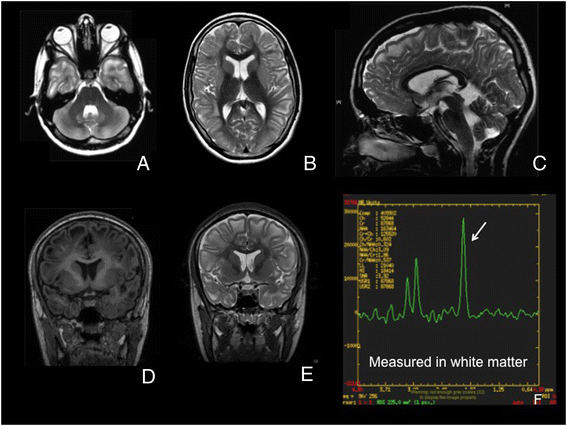

Supplement: Supplementary file 2 — Authors’ original file for figure 1 [file 40734_2014_9_MOESM2_ESM.gif]

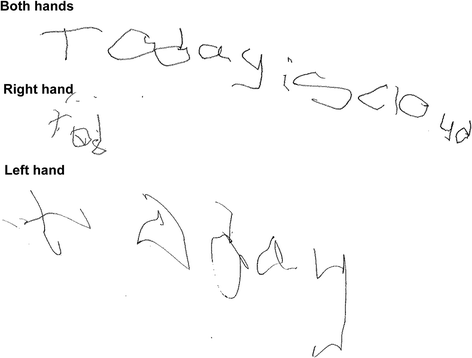

Supplement: Supplementary file 3 — Authors’ original file for figure 2 [file 40734_2014_9_MOESM3_ESM.gif]
